# Swept-source optical coherence tomography and swept-source optical coherence tomography angiography findings in circumscribed choroidal hemangioma before and after transpupillary thermotherapy

**DOI:** 10.1007/s10103-024-04088-x

**Published:** 2024-06-05

**Authors:** Ibadulla Mirzayev, Ahmet Kaan Gündüz, Ahmet Ergin

**Affiliations:** 1 Department of Ophthalmology, Ankara University Faculty of Medicine, Ankara, Turkey; 2 Halil Şıvgın Çubuk State Hospital, Ophthalmology Clinic, Ankara, Turkey; 3 Private Eye Clinic, Farilya Business Center 8/38, Ufuk Universitesi Cad, Çukurambar, Ankara, 06510 Turkey; 4 Department of Ophthalmology, Balıkesir University Faculty of Medicine, Balıkesir, Turkey

**Keywords:** Circumscribed choroidal hemangioma, Indocyanine green-enhanced transpupillary thermotherapy, Internal tumor vessels, Swept-source optical coherence tomography angiography, Transpupillary thermotherapy

## Abstract

**Purpose:**

To investigate the swept-source optical coherence tomography (SS-OCT) and SS-OCT angiography (SS-OCTA) findings in circumscribed choroidal hemangioma (CCH) before and after treatment with transpupillary thermotherapy (TTT).

**Methods:**

The clinical records of 21 eyes having CCH imaged with SS-OCT/SS-OCTA between September 2018 and December 2022 were evaluated.

**Results:**

SS-OCT examination in CCH showed dome-shaped appearance (100%), choroidal shadowing (100%), expansion of choroidal structures (100%), subretinal fluid (66.7%), intraretinal edema/schisis (33.3%), retinal pigment epithelium (RPE) atrophy (19.0%), hyperreflective dots (19.0%), and epiretinal membrane (4.8%). Internal arborizing tumor vessels showing hyperreflectivity were observed in the choriocapillaris slab on SS-OCTA in all eyes. In the deep capillary plexus (DCP), flow void changes were seen in 7 eyes with intraretinal schisis/cystoid macular edema. Four CCHs > 2 mm in thickness showed outer retinal involvement due to unmasking of flow in intratumoral vessels related to RPE atrophy. Following TTT/indocyanine green-enhanced TTT (ICG-TTT) of CCH, SS-OCT findings included total/partial resolution of subretinal fluid (57.1%), complete/partial regression of the tumor (52.4%), and RPE atrophy (33.3%). After treatment; loss of choriocapillaris, decrease in tumor vascularity together with increase in the fibrous component and flow void areas were detected on SS-OCTA.

**Conclusions:**

SS-OCT/SS-OCTA are useful non-invasive tools for imaging the structural/vascular changes in CCHs managed with TTT or ICG-TTT. On SS-OCTA, hyporeflective spaces localizing to edema/schisis in the DCP and arborizing tumor vessels within a hyporeflective stromal background in the choriocapillaris slab were observed. After TTT/ICG-TTT, a decrease in tumor vessels and an increase in the fibrous component and flow-void areas inside the CCH were detected on SS-OCTA.

## Introduction

Choroidal hemangioma is the most common benign vascular tumor of the choroid and can be circumscribed or diffuse [1]. Diffuse choroidal hemangioma usually occurs in the setting of Sturge-Weber syndrome. Circumscribed choroidal hemangioma (CCH) is sporadic and not accompanied by systemic findings.

Due to the longer wavelength (1050 nm) employed and high A-scan rates (100.000 A-scan/s), swept-source optical coherence tomography (SS-OCT) achieves greater tissue penetration and allows better visualization of the internal configuration of the tumors compared to spectral-domain (SD) OCT systems [2–4]. OCT angiography (OCTA) is a novel non-invasive procedure for evaluating retinal and choroidal vascular tissues [2, 5]. OCTA provides the opportunity for segmentation of the retina into specific layers which is not possible using traditional dye-based angiography systems. OCTA may be a useful method for evaluation of vascular structures and response of these structures to treatment in CCH cases [6, 7].

Transpupillary thermotherapy (TTT) is usually used for CCHs with largest tumor base diameter of ˂10 mm, thickness of ˂4 mm, and shallow subretinal fluid (SRF) [8]. TTT rises tissue temperature > 42 < 60 °C. This is below the coagulation necrosis level achieved with laser photocoagulation [9, 10]. At this temperature level, cytolysis, ischemic necrosis, and shrinkage of the tumor occur [9–11]. Indocyanine green is a protein-bound photosensitizing agent that increases the effects of TTT through greater absorption at the level of choroid leading to occlusion and thrombosis of the vascular channels [12, 13]. The indications for TTT also apply to indocyanine green-enhanced TTT (ICG-TTT).

There have been few reports in the literature investigating SS-OCT and SS-OCTA findings in a limited number of CCH cases [4, 6, 7, 14–17]. Herein, we report the SS-OCT/SS-OCTA findings in 21 CCH cases.

## Materials and methods

The clinical records of 21 CCH cases imaged with SS-OCT and SS-OCTA (Topcon DR1 Triton Plus, Tokyo, Japan) between September 2018 and December 2022 were retrospectively reviewed. The parameters that were assessed included patient age, gender, symptoms, duration of symptoms, baseline visual acuity (VA), quadrant location of tumor epicenter (extrafoveolar macula, fovea, superior, inferior, temporal, and nasal), largest tumor base diameter (LTBD), tumor thickness, tumor distance to foveola and optic disc, SRF, retinoschisis, retinal pigment epithelium (RPE) hyperplasia, RPE fibrous metaplasia, cystoid macular edema, epiretinal membrane, orange pigment, treatment methods, and treatment complications. The study adhered to the tenets of the Helsinki Declaration and all patients signed informed consent form. All participants included in the study provided informed consent for publication of individual person’s data in any form.The Ethics Committee of Ankara University Faculty of Medicine approved the study (Approval number: İ1-45-21).

Multimodal imaging with color fundus photography, fundus autofluorescence (FAF), SS-OCT, SS-OCTA, and fluorescein angiography (FA, in selected cases) were also done using Triton Plus machine. For SS-OCT imaging, 6, 9, or 12 mm scans were employed. A- and B mode ultrasonography were performed using Ellex Eye Cubed device (Mawson Lakes, Australia).

### SS-OCT and SS-OCTA imaging

SS-OCT features evaluated were tumor shape and reflectivity, presence of choroidal shadowing, presence of SRF, intraretinal edema/schisis, RPE and/or outer retinal atrophy, hyperreflective dots, and epiretinal membrane. Among the SS-OCTA features evaluated were tumor margins (well-defined vs ill-defined), internal reflectivity, superficial capillary plexus (SCP), deep capillary plexus (DCP), outer retina, choriocapillaris, presence of intrinsic tumor vessels, choroidal vascular findings, and presence of choroidal neovascularization. The automated segmentation for SS-OCTA slabs were as follows: (1) The SCP slab: from 2.6 µ beneath the internal limiting membrane to 15.6 µ beneath the interface of inner plexiform layer (IPL) and inner nuclear layer (INL); (2) The DCP slab from 15.6 µ beneath the IPL/INL to 70.2 µ beneath IPL/INL; (3) The outer retina-from 70.2 µ below the IPL/INL border to Bruch’s membrane (BM); (4) The choriocapillaris-from BM to 10.4 µ below BM. The slab thicknesses may be changed to reveal the tumor features in the area of interest. SS-OCT and SS-OCTA mages were evaluated by two different authors (AKG and IM).

### TTT and ICG-TTT methods

Transpupillary thermotherapy was performed using Volk Area Centralis Laser Lens (Volk Optical Inc, Mentor, Ohio, USA) and diode laser emitting at 810 nm with a slit-lamp biomicroscope delivery system (Oculight SLx; Iridex Corp., Mountain View, CA, USA). Spot diameter was 2–3 mm. The beginning power was set at 200–300 mW and was increased in 50–100 mW increments until a light gray color change was observed on the tumor surface during the second half of the 1 min application period. The power range used in this study was 200–600 mW. Each spot was maintained for 1 min on the tumor surface to ensure that TTT achieved the necessary temperature raise in the treated tissue. The exposure time was adjusted to cover the entire tumor surface in an overlapping fashion, with larger tumors requiring longer exposure times. The total exposure time was 5–8 min. Subsequent TTT sessions were applied in cases where resolution of SRF was not observed after the first session. The number of sessions for TTT ranged from 1 to 4.

In cases treated with ICG-TTT, 25 mg/5 ml intravenous ICG (ICG-Pulsion, Pulsion Medical Systems, Munich, Germany) was injected 2 min before starting treatment. All other application steps were same as outlined above for TTT. The number of sessions ranged from 1 to 4 based on the resolution of SRF.

The patients were counselled on the potential enhanced therapeutic effect of ICG-TTT vs. TTT. Those who were willing to made a copayment for ICG were treated using ICG-TTT.

Multimodal imaging was done before and each visit after treatment. Tumor regression was evaluated based on RECIST criteria although CCH is not really a solid tumor [18]. Based on RECIST criteria complete regression was defined as disappearance of the tumor, partial regression is defined as at least a ≥ 30% decrease in thickness, and stable disease corresponds to shrinkage of less than 30% in tumor thickness. Additionally, we also considered disappearance of SRF as an end-point in treatment.

### Statistical analysis

SPSS for Windows version 11.5 (SPSS Inc, Chicago, IL, USA) was used. Variables were expressed as mean/median (minimum-maximum). Also, categorical variables were described as counts (percentages). Associations between categorical variables were determined by Pearson Chi-Square/Fisher Exact Test.

## Results

**Fig. 1:**
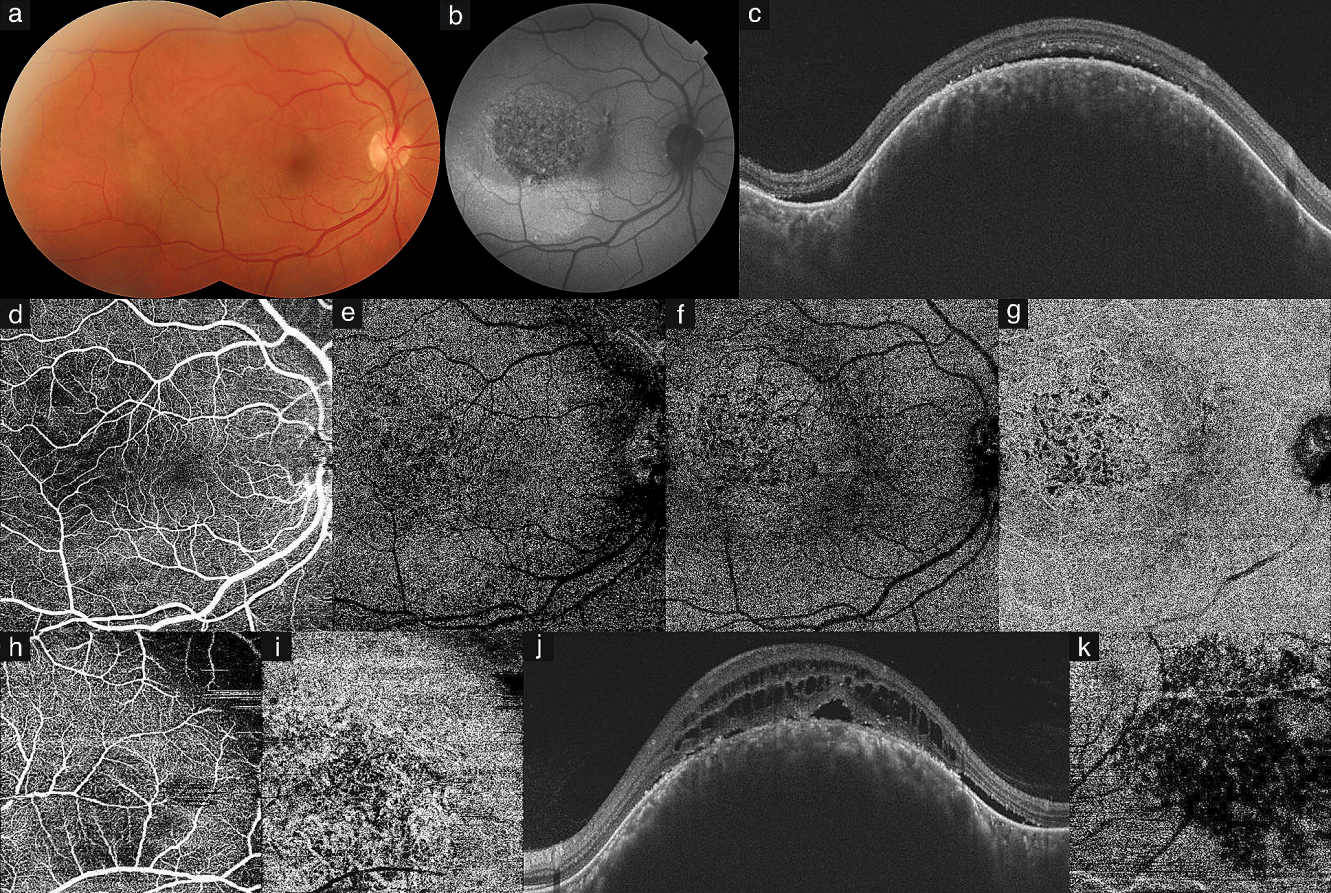
Pretreatment findings in circumscribed choroidal hemangioma (CCH). **Eye #1 (a)** Color composite photograph of CCH located temporally in the right eye. **(b)** Fundus autofluorescence (AF) shows patchy AF in CCH and hyperAF from subretinal fluid below the tumor. **(c)** Swept-source optical coherence tomography (SS-OCT) demonstrates dome-shaped medium reflective CCH, posterior shadowing, and presence of subretinal fluid. **(d-g)** SS-OCT angiography (SS-OCTA) shows normal superficial capillary plexus (SCP) **(d)** and deep capillary plexus (DCP) **(e)**, outer retinal involvement due to unmasking of flow in intratumoral vessels from retina pigment epithelial atrophy **(f)**, and intermingled club-shaped anastomosing intratumoral vessels at the choroidal slab **(g)**. **Eye #2 (h-i)** SS-OCTA depicts pressure vascular changes in the SCP **(h)** and intratumoral vessels in the choriocapillaris **(i). Eye #3 (j)** SS-OCT shows dome-shaped medium reflective CCH, hyperreflective foci, intraretinal schisis, retinal pigment epithelial atrophy, and subretinal fluid. **(k)** SS-OCTA of the same eye demonstrates flow void areas from intraretinal schisis in the DCP

**Fig. 2:**
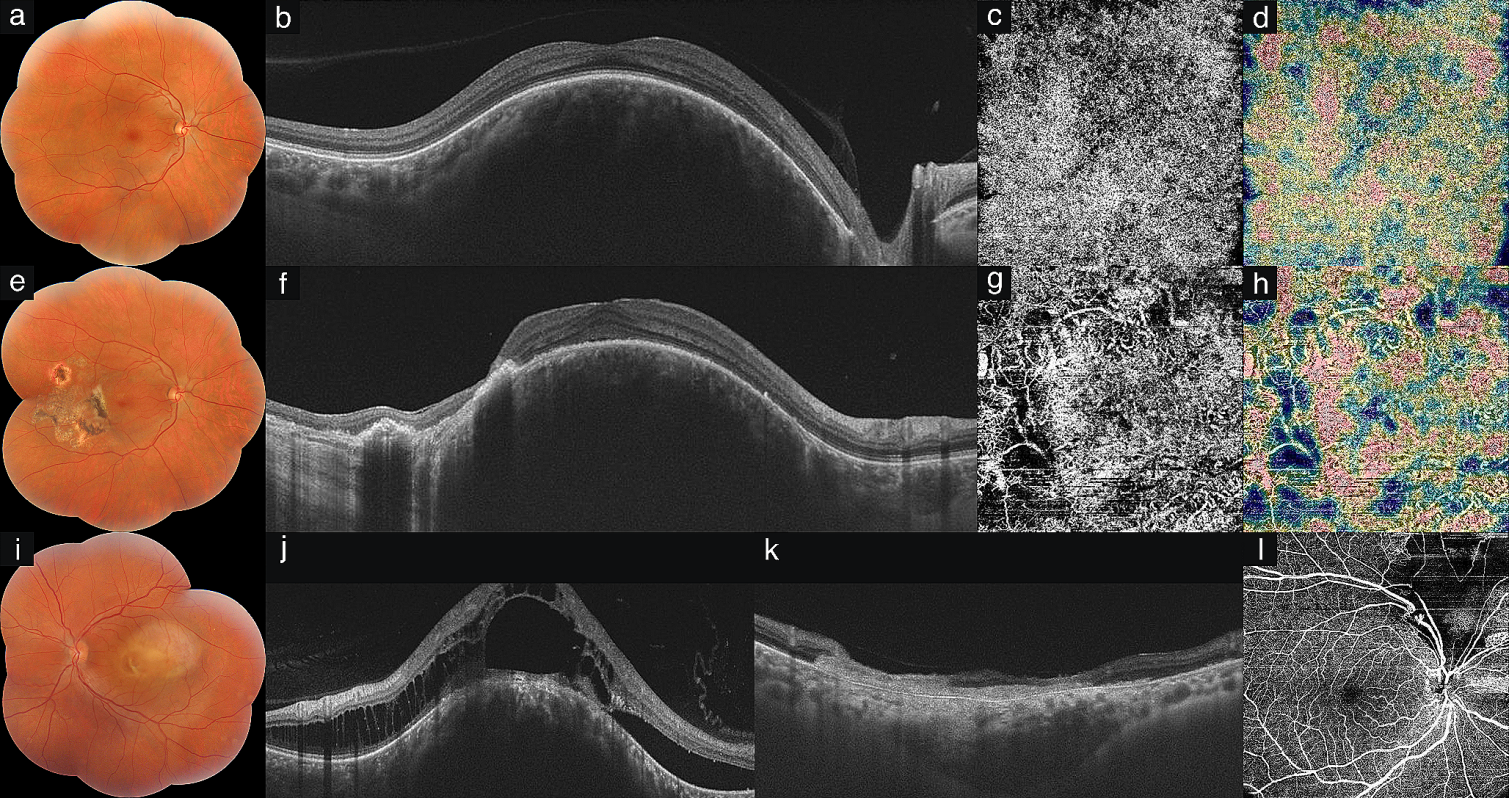
Pre- and posttreatment findings in circumscribed choroidal hemangioma (CCH). **Eye #4** Figures a-d pertain to the initial examination. **(a)** Color composite photograph shows subfoveal CCH in the right eye. **(b)** Swept-source optical coherence tomography (SS-OCT) demonstrates dome-shaped medium reflective CCH. **(c)** SS-OCT angiography (SS-OCTA) shows irregular vascular network at the choriocapillaris slab. **(d)** The vascular density map of choriocapillaris demonstrates increased density corresponding to the tumor location. Figures e-h refer to the final examination at 2 years follow-up after 3 sessions of indocyanine green-enhanced transpupillary thermotherapy (ICG-TTT). **(e)** Color composite photograph shows areas of retinal pigment epithelial atrophy after treatment. **(f)** SS-OCT depicts retinal pigment epithelial atrophy and increased light transmission into the choroid. **(g)** SS-OCTA shows a decrease in the density of vessels at the choroidal slab. **(h)** The vascular density map demonstrates decrease in density manifesting as cool colors (light green and blue) compared to first examination at the choriocapillaris slab. **Eye #5 (i)** Color composite photograph shows of macular CCH in the left eye. **(j)** Pretreatment SS-OCT shows dome-shaped medium reflective CCH, intraretinal schisis, retinal pigment epithelial atrophy, and subretinal fluid. **(k)** SS-OCT depicts total regression of the tumor, retinal pigment epithelial and outer retinal atrophy, and visibility of Bruch’s membrane after 2 sessions of TTT. **Eye #6 (l)** SS-OCTA shows hypoperfusion areas in the superficial capillary plexus consistent with retinal atrophy after 4 sessions of ICG-TTT.

Patient demographics, tumor features, ultrasonography, and FAF findings are given in Table 1. Thirteen (61.9%) patients were male and 8 (38.9%) were female. The mean age at diagnosis was 47.9 years. Symptoms were decreased vision in 19 (90.5%) patients and flashing lights in 2 (9.5%). The mean time elapsed from initial symptoms till diagnosis was 13.9 (median 8, range 1–48) months. At presentation, VA was ≥ 20/40 in 8 (38.1%) eyes, < 20/40->20/200 in 2 (9.5%), and ≤ 20/200 in 11 (52.4%). The most frequent tumor location was macula (9 eyes, 42.9%) eyes (Figs. 1a, 2a, e, and i). The mean LTBD and the mean tumor thickness were 6.5 and 2.0 mm, respectively.

**Table 1:** Patient demographics, tumor features, ultrasonography, and fundus autofluorescence findings in eyes with circumscribed choroidal hemangioma

	Circumscribed choroidal hemangioma (*n*=21)
**Mean patient age (median, range)**	47.9 (47, 22-82)
**Gender, n (%)**	
Female	8 (38.9)
Male	13 (61.9)
**Eye, n (%)**	
Right	9 (42.9)
Left	12 (57.1)
**Color, n (%)**	
Orange yellow	21 (100)
**Tumor location, n (%)**	
Macula (extrafoveolar)	7 (33.3)
Fovea	2 (9.5)
Superior	3 (14.3)
Inferior	5 (23.8)
Temporal	3 (14.3)
Nasal	1 (4.8)
**Distance to foveola, mean (median, range), mm**	1.5 (1.0-5)
**Distance to optic disc, mean (median, range), mm**	2.7 (2.0-8)
**LTBD, mean (median, range), mm**	6.5 (6.5, 3-10)
**Tumor thickness, mean (median, range), mm**	2.2 (2.2, 2.0-3.3)
**Accompanying feature, n (%)**	
Subretinal fluid	14 (66.7)
Retinoschisis	7 (33.3)
Orange pigment	2 (9.5)
RPE atrophy	4 (19.0)
Epiretinal membrane	1 (4.8)
**B-mode ultrasonography, n (%)**	
Acoustically solid	21 (100)
**AF, n (%)**	
Pachy hyperAF	14 (66.7)
IsoAF	4 (19.0)
Diffuse hyperAF	2 (9.5)
Pinpoint hyperAF	1 (4.8)

LTBD: Largest tumor base diameter; AF: Autofluorescence

All CCHs were acoustically solid on B-mode ultrasonography. The most common appearance of CCHs on FAF was patchy autofluorescence (14 eyes, 66.7%) (Fig. 1b). SS-OCT and SS-OCTA features of CCHs are given in Table 2. SS-OCT showed dome-shaped appearance, choroidal shadowing, and expansion of choroidal structures in all eyes (100%) (Figs. 1c, j, 2b, and j). Of 21 CCHs, 17 (81.0%) demonstrated medium and 4 (19.0%) demonstrated low reflectivity. SRF, intraretinal edema/schisis, RPE and/or outer retinal atrophy, hyperreflective dots, and epiretinal membrane were seen in 14 (66.7%), 7  (33.3%), 4 (19.0%), 4 (19.0%), and 1 (4.8%) eyes, respectively (Figs. 1j and 2j).

**Table 2:** Swept-source optical coherence tomography and swept-source optical coherence tomography angiography findings in 21 eyes with circumscribed choroidal hemangioma at baseline

	Circumscribed choroidal hemangioma (*n* = 21)
**Swept-source optical coherence tomography**	
**Tumor shape,*n*(%)**	
Dome	21 (100)
** Tumor reflectivity, n (%)**	
High	-
Medium	17 (81.0)
Low	4 (19.0)
** Choroidal shadowing, n (%)**	21 (100)
** Choroidal vascular structures, n (%)**	
Expansion	21 (100)
** Retina**	
Subretinal fluid	14 (66.7)
Intraretinal edema/schisis	7 (33.3)
RPE and/or outer retinal atrophy*	4 (19.0)
Hyperreflective dots**	4 (19.0)
Epiretinal membrane	1 (4.8)
**Swept-source optical coherence tomography angiography**	
** Tumor margins, n (%)**	
Well-defined	19 (90.5)
Ill-defined	2 (9.5)
** Internal reflectivity, n (%)**	
Mixed hypo and hyperreflective	21 (100)
** Superficial and deep capillary plexi**	
** Pressure vascular changes, n (%)**	
Present	3 (14.3)
Absent	18 (85.7)
** Flow-void areas, n (%)**	
Present	7 (33.3)
Absent	14 (66.7)
** Outer retinal involvement, n (%)**	
Present	4 (19.0)
Absent	17 (81.0)
** Choriocapillaris, n (%)**	
Present	21 (100)
** Intrinsic tumor vessels, n (%)**	
Visible	21 (100)
** Choroidal neovascularization**	
Absent	21 (100)

RPE: Retinal pigment epithelium. *: Atrophic changes in the RPE and outer retina including photoreceptors, ellipsoid zone, external limiting membrane, outer nuclear layer and outer plexiform layer; **: Subretinal or intraretinal lipofuscin collections

All CCHs except two presented with well-defined borders. There was no tumor involvement in SCP and DCP (Figs. 1d and e). Three (14.3%) CCHs with thickness > 3 mm demonstrated pressure-induced vascular changes in the SCP and DCP (Fig. 1h). In DCP, flow-void areas from intraretinal schisis/edema were observed in 7/21 (33.3%) eyes (Fig. 1k). Four (19.0%) CCHs > 2 mm in thickness showed outer retinal involvement due to unmasking of flow in intratumoral vessels related to RPE atrophy (Fig. 1f). Internal arborizing tumor vessels resembling noodle-like or spaghetti-like pattern showing hyperreflectivity were detected in the choriocapillaris slab in all eyes (Figs. 1g, i, and 2c). B-scan angiography overlay and vascular density map showed increased flow signals over the tumor in all eyes (Fig. 2d).

TTT was done in 5 (23.8%) eyes and ICG-TTT in 16 (76.2%). There were no statistically significant differences between TTT and ICG-TTT groups with respect to initial visual acuity and tumor features including tumor location, tumor distance to foveola, distance to optic disk, LTBD, tumor thickness, SRF, and others. SRF was found in 3/5 (60.0%) eyes and in 11/16 (68.8%) eyes treated with TTT and ICG-TTT, respectively. Of 5 eyes undergoing TTT, 1 (20.0%) eye received 1 treatment session, 2 (40.0%) eyes received 2 sessions, and 2 (40.0%) eyes received 4 sessions. Among 16 eyes managed with ICG-TTT, 1 (6.3%) eye received 1 treatment, 8 (50.0%) eyes received 2 treatments, 6 (37.5%) eyes received 3 treatments, and 1 (6.3%) eye received 4 treatments.

The mean follow-up was 30.8 (median 20, range 3-160) months. There were no statistically significant differences between 2 treatment groups in terms of the last VA ≥ 20/40, VA improvement by ≥ 2 lines, resolution of SRF, and decrease in tumor thickness by ultrasonography ≥ 30%. The last VA was ≥ 20/40 in 13 (61.9%) eyes, < 20/40->20/200 in 3 (14.3%), and ≤ 20/200 in 5 (23.8%). VA improvement by ≥ 2 lines was found in 11 (52.4%) eyes. VA improvement by ≥ 2 lines was detected in 1/5 (20.0%) eyes undergoing TTT and in 10/16 (64.0%) eyes treated with ICG-TTT (*p* > 0.05). SRF resolved completely in 8/14 (57.1%) eyes and persisted in 6/14 (42.9%) eyes. Resolution of SRF was seen in 1/3 (33.3%) eyes managed with TTT and in 7/11 (63.6%) eyes undergoing ICG-TTT (*p* > 0.05). The eye with SRF resolution following TTT had received 2 treatment sessions. The mean number of treatment sessions in eyes with SRF resolution after ICG-TTT was 1.9. The mean number of treatment sessions in eyes having persistent SRF after TTT and ICG-TTT was 3.0 and 2.5, respectively. Due to the limited number of eyes in the TTT group, statistical analysis could not be done regarding the relationship between the number of sessions and SRF resolution. Complete disappearance, decrease in tumor thickness ≥ 30%, and decrease in tumor thickness < 30% were found in 3 eyes (14.3%), 6 eyes (28.6%), and 12 eyes (57.1%), respectively. Decrease in tumor thickness ≥ 30%/complete dissapearance was noted in 9/21 (42.9%), 2/5 (40.0%), and 7/16 (43.8%) eyes in the whole study group, TTT group, and ICG-TTT group, respectively (*p* > 0.05). Complications of treatment were intraretinal hemorrhage in 2 (9.5%) eyes and branch retinal vein occlusion in 1 (4.8%) eye. Following TTT/ICG-TTT, SS-OCT revealed decrease in tumor thickness (52.4%), partial or total regression of SRF and intraretinal edema (57.1%), RPE atrophy (33.3%), increased posterior light transmission (19.0%), and loss of lamination and atrophy of retina (19.0%) (Figs. 2f and k). On SS-OCTA, a decrease in intratumoral vessels in the choriocapillaris slab was observed following treatment in all eyes (Fig. 2g). In all eyes, loss of choriocapillaris was also detected. B-scan angiography overlay and vascular density map demonstrated decrease in flow signals compared to the initial examination (Fig. 2h). There was also an increase in flow void areas in these eyes consistent with enlargement of non-vascular areas (Figs. 2g and 2l).

## Discussion

Although CCH is usually diagnosed ophthalmoscopically, ultrasonography, FAF, OCT, FA, and ICGA are also used as ancillary tests to verify tumor diagnosis and assess treatment response. In recent years, OCTA has also used in the diagnosis of CCH and in the evaluation of treatment results [3, 6, 7]. In this study, we focused on SS-OCT/SS-OCTA findings in CCHs managed with TTT/ICG-TTT.

On SS-OCT, CCHs demonstrated a dome-shaped (100%) appearance with medium (81.0%) or low (19.0%) reflectivity, choroidal shadowing (100%), SRF (66.7%), intraretinal edema/intraretinal schisis (33.3%), RPE and or outer retinal atrophy (19.0%), hyperreflective dots (19.0%), and epiretinal membrane (4.8%). In addition, there was expansion of medium and large sized choroidal vessels without compression of choriocapillaris (100%).

On OCTA, there were no structural changes in SCP and DCP slabs in most of the eyes with CCH. However, pressure vascular changes were noted in 14.3% of eyes in the SCP and DCP. Flow-void areas from intraretinal schisis/edema were detected in 33.3% of eyes in the DCP. Similar findings in the DCP have been reported before [3]. In a study comparing eyes with CCH and normal fellow eyes by OCTA, there was no difference in the SCP foveal avascular zone (FAZ), DCP FAZ, and SCP vascular density. However, the mean DCP vascular density was decreased in eyes harboring CCH [19]. Subgroup analysis in the same study demonstrated that eyes with previous/current cystoid macular edema/SRF had decreased DCP vascular density, while eyes without previous/current cystoid macular edema/SRF had similar DCP vascular density compared with fellow eyes [19]. The authors also emphasized that the decrease in DCP vascular density from cystoid macular edema/SRF was not likely a result of artifact as all images were individually analyzed for accuracy in segmentation and only with good signal strengths were included [19].

In 44.4% of CCHs, RPE atrophy was detected over the tumor. In these eyes, outer retinal involvement was observed due to unmasking of flow in intratumoral vessels. Cennamo et al. also noted a dense irregular vascular network in the outer retina in 6 eyes with CCH [16].

On SS-OCTA, we detected internal arborizing tumor vessels demonstrating hyperreflectivity in the choroidal slab in all eyes with CCH. Similarly, superficial multiple whitish irregular vessels resembling “bag of worms” and deeper vessels demonstrating “club-like” appearance were demonstrated at varying depths at the choroidal slab in previous studies [14]. Some authors identified whitish, tortuous, bending or curved vessels, interlaced with few intervening signal void areas on SS-OCTA images of CCH and resembled this appearance to a noodle like-pattern [20, 21]. The transition from irregularly shaped tumor vessels to regularly shaped normal vessels at the tumor margin was also demonstrated [14].

In our study, B-scan angiography overlay demonstrated increased flow signals over the tumor in all eyes. Similarly, Toledo et al. reported high vascular flux over CCHs in B-scan overlay [15]. Quiescent tumors with few intratumoral vessels were reported to remain stable [22]. In a case in which intratumoral blood flow was not detected on OCTA, no change was found in VA and SRF at the 6-month follow-up without treatment [22]. This shows that OCTA can also be useful in monitoring CCH cases that are observed.

SS-OCT findings after TTT and ICG-TTT of CCHs included decrease in tumor thickness, partial or total resolution of SRF and intraretinal edema, RPE atrophy, increased posterior light transmission, and retinal atrophy with loss of lamination. SS-OCT provides invaluable information on retinal changes including retinoschisis overlying the tumor and regression of intraretinal edema and SRF following treatment.

We noticed a decrease in intratumoral vessels following TTT/ICG on SS-OCTA. Giudice et al. reported that two days after photodynamic therapy (PDT), large intralesional vascular channels of CCH appeared dark on OCTA [7]. Cennamo et al. noted reduction of the vessel and flow areas between baseline and 1 year follow-up in 7 CCH cases treated with Ruthenium-106 brachytherapy [23]. The authors speculated that this result was due to the vaso-occlusive effects of PDT and brachytherapy which causes thrombosis of angiomatous channels [7, 23]. However, in another study, persistent medium-sized crisscrossing vessels over the tumor following 2–3 sessions of laser photocoagulation have been reported on OCTA [17].

In our study, loss of choriocapillaris and an increase in the fibrous component and flow void areas were observed on SS-OCTA after TTT/ICG-TTT. A study evaluating comparative OCTA findings before and after TTT (3 eyes) or PDT (4 eyes) reported that complete loss of choriocapillaris and absence of deeper choroidal vessels was observed in all TTT-treated eyes. In contrast, patency of choriocapillaris (100%) and persistence of deeper choroidal vessels (50%) were found in PDT-treated eyes [6]. Partial signal void areas were demonstrated in both groups after treatment (100%) [6]. In terms of preserving choroidal vasculature, the authors recommended PDT in CCH, particularly in cases involving the fovea [6]. TTT/ICG-TTT should be used carefully in subfoveal CCHs [24, 25]. A foveal sparing approach is necessary for subfoveal tumors when using TTT for tumors at this location.

In our series, VA improvement by ≥ 2 lines was observed in 52.4% of eyes after treatment with TTT/ICG-TTT. Complete resolution of SRF was seen in 57.1% of eyes. Although statistical analysis could not be performed due to the limited number of eyes in the TTT group, it appears that fewer treatment sessions of ICG-TTT may be required for SRF resolution compared to TTT. Complete disappearance, decrease in tumor thickness ≥ 30%, and decrease in tumor thickness < 30% were noted in 14.3%, 28.6%, and 57.1% of eyes, respectively. Rate of VA improvement by ≥ 2 lines and rate of complete resolution of SRF were higher in ICG-TTT group compared to TTT group, while rate of decrease in tumor thickness ≥ 30% was higher in TTT group compared to ICG-TTT group. However, these differences were not statistically significant. Our detailed comparative results of laser treatment modalities used in CCH have been reported previously [25].

Our study has certain limitations. First, it is retrospective. Second, patient sample was relatively small. Third, the limited number of patients in the TTT group compared to the ICG-TTT group prevented a more reliable statistical evaluation among the treatment groups.

In summary, SS-OCT and SS-OCTA are useful non-invasive methods for evaluating structural and vascular changes in CCH cases before and after treatment. CCHs usually demonstrate a dome-shaped appearance, choroidal shadowing, and expansion of choroidal structures on SS-OCT. On SS-OCTA, CCHs are characterized by well-defined margins, a mixed reflective internal tumor structure featuring intertwined and irregularly organized large-calibre vessels mixed with signal void areas, presumably representing connective tissue components of the tumor. After TTT/ICG-TTT, RPE atrophy, increased light transmission into the choroid, and decrease in tumor thickness were noted on SS-OCT. Decrease in tumor vessels and increase in the fibrous component and flow-void areas of the tumor were detected on SS-OCTA following TTT/ICG-TTT.
